# Transcriptome-wide N6-methyladenosine (m^6^A) methylation in watermelon under CGMMV infection

**DOI:** 10.1186/s12870-021-03289-8

**Published:** 2021-11-08

**Authors:** Yanjun He, Lili Li, Yixiu Yao, Yulin Li, Huiqing Zhang, Min Fan

**Affiliations:** Institute of Vegetables, Zhejiang Academy of Agricultural Sciences, Hangzhou, 310021 Zhejiang China

**Keywords:** Watermelon, *Cucumber green mottle mosaic virus* (CGMMV), m^6^A methylation, m^6^A-seq, RNA-seq

## Abstract

**Background:**

*Cucumber green mottle mosaic virus* (CGMMV) causes substantial global losses in cucurbit crops, especially watermelon. N6-methyladenosine (m^6^A) methylation in RNA is one of the most important post-transcriptional modification mechanisms in eukaryotes. It has been shown to have important regulatory functions in some model plants, but there has been no research regarding m^6^A modifications in watermelon.

**Results:**

We measured the global m^6^A level in resistant watermelon after CGMMV infection using a colorimetric method. And the results found that the global m^6^A level significantly decreased in resistant watermelon after CGMMV infection. Specifically, m^6^A libraries were constructed for the resistant watermelon leaves collected 48 h after CGMMV infection and the whole-genome m^6^A-seq were carried out. Numerous m^6^A modified peaks were identified from CGMMV-infected and control (uninfected) samples. The modification distributions and motifs of these m^6^A peaks were highly conserved in watermelon transcripts but the modification was more abundant than in other reported crop plants. In early response to CGMMV infection, 422 differentially methylated genes (DMGs) were identified, most of which were hypomethylated, and probably associated with the increased expression of watermelon m^6^A demethylase gene *ClALKBH4B*. Gene Ontology (GO) analysis indicated quite a few DMGs were involved in RNA biology and stress responsive pathways. Combined with RNA-seq analysis, there was generally a negative correlation between m^6^A RNA methylation and transcript level in the watermelon transcriptome. Both the m^6^A methylation and transcript levels of 59 modified genes significantly changed in response to CGMMV infection and some were involved in plant immunity.

**Conclusions:**

Our study represents the first comprehensive characterization of m^6^A patterns in the watermelon transcriptome and helps to clarify the roles and regulatory mechanisms of m^6^A modification in watermelon in early responses to CGMMV.

**Supplementary Information:**

The online version contains supplementary material available at 10.1186/s12870-021-03289-8.

## Background

N6-methyladenosine (m^6^A) methylation is the most prevalent internal modifier of eukaryote RNA and is widely present. It plays multiple roles in RNA metabolism, including stability and degradation [1], alternative splicing [2], translation efficiency [3], and nuclear export [4]. In plants, the dynamic patterning of m^6^A modifications in mRNA is mediated by three clades of elements, including m^6^A writer, eraser, and reader proteins [5]. The m^6^A methylation is formed by the m^6^A methyltransferase writer, which is complex composed of N6-adenosine-methyltransferase MT-A70-like (MT) proteins MTA, MTB, and other factors. In Arabidopsis, removal of m^6^A is catalyzed by m^6^A demethylases of alkylated DNA repair proteins AlkB homologs (ALKBHs) such as ALKBH9B and ALKBH10B. The discovery of methyltransferases and demethylases implies the m^6^A modification is a reversible and dynamic RNA methylation. A clade of proteins containing YTH-domain serves as m^6^A readers such as Arabidopsis EVOLUTIONARILY CONSERVED C-TERMINAL REGION2/3/4 (ECT2/3/4) and Cleavage and Polyadenylation Specificity Factor 30 (CPSF30) proteins, which could bind specifically to m^6^A sites (such as RRACH) and mediate specific functions.

Evidence indicates that m^6^A RNA methylation is highly conserved in plants and involved in multiple developmental and biological processes, such as embryonic development [6, 7], trichome branch development [8–10], leaf initiation [11], flowering and root development [12]. Moreover, a few reports suggest that m^6^A methylation plays important roles in plant antiviral immunity. For example, Arabidopsis m^6^A demethylase ALKBH9B can remove m^6^A modifications from viral RNA and repress *Alfalfa mosaic virus* (AMV) infections [13]. In tobacco, the global m^6^A level was reduced under *Tobacco mosaic virus* (TMV) infection, probably associated with decreased m^6^A methyltransferase and increased demethylase expression [14]. Furthermore, m^6^A levels in susceptible rice increased in response to *Rice black-streaked dwarf virus* (RBSDV) and *Rice stripe virus* (RSV) infection, as m^6^A methylation mainly depends on genes which are not actively expressed in virus-infected rice plants via m^6^A-seq analyses [15]. In wheat, the transcriptome-wide m^6^A profile of two varieties with different resistance to *Wheat yellow mosaic virus* (WYMV) revealed that many genes related to plant defense responses and plant-pathogen interaction significantly changed in both m^6^A and mRNA levels [16]. Overall, these studies suggested that m^6^A modification plays important roles in plant response to viral infection, but the relevant regulatory mechanism is complex and still needs to be elucidated.

*Cucumber green mottle mosaic virus* (CGMMV), a member of the genus *Tobamovirus*, has spread rapidly and caused huge economic losses in cucurbits especially watermelon. Consequently, it is important to determine host response mechanisms of watermelon to CGMMV. Previously, the transcriptome, and proteomic, microRNA (miRNA), and virus derived small interfering RNAs (vsiRNAs) profiles via high-throughput sequencing have been performed, and some CGMMV-responsive genes, miRNAs, and vsiRNAs were identified in watermelon [17–19]. However, as the most important post-transcriptional mechanism, m^6^A modification profiles in watermelon following CGMMV infection have not been explored.

In this study, we found that the global m^6^A level in resistant watermelon clearly decreased after CGMMV infection by colorimetry. Therefore, we analyzed watermelon m^6^A methylation profiles in response to CGMMV infection via whole-genome m^6^A-seq and bioinformatics approaches. Meanwhile, combined with RNA-sequencing, the response patterns and putative functions of differentially expressed m^6^A-modified genes (DMGs) derived from the transcriptomes of CGMMV-infected watermelon leaves were analyzed. Furthermore, we verified some target genes were verified by m^6^A-immunoprecipitation (IP)-qPCR (m^6^A-IP-qPCR). Our study represents the first comprehensive characterization of m^6^A modification in the watermelon transcriptome. Furthermore, we attempted to elucidate the potential m^6^A-mediated roles and regulatory mechanisms in watermelon responses to CGMMV.

## Results

### CGMMV infection decreased the m^6^A global level in resistant watermelon plants

**Fig. 1:**
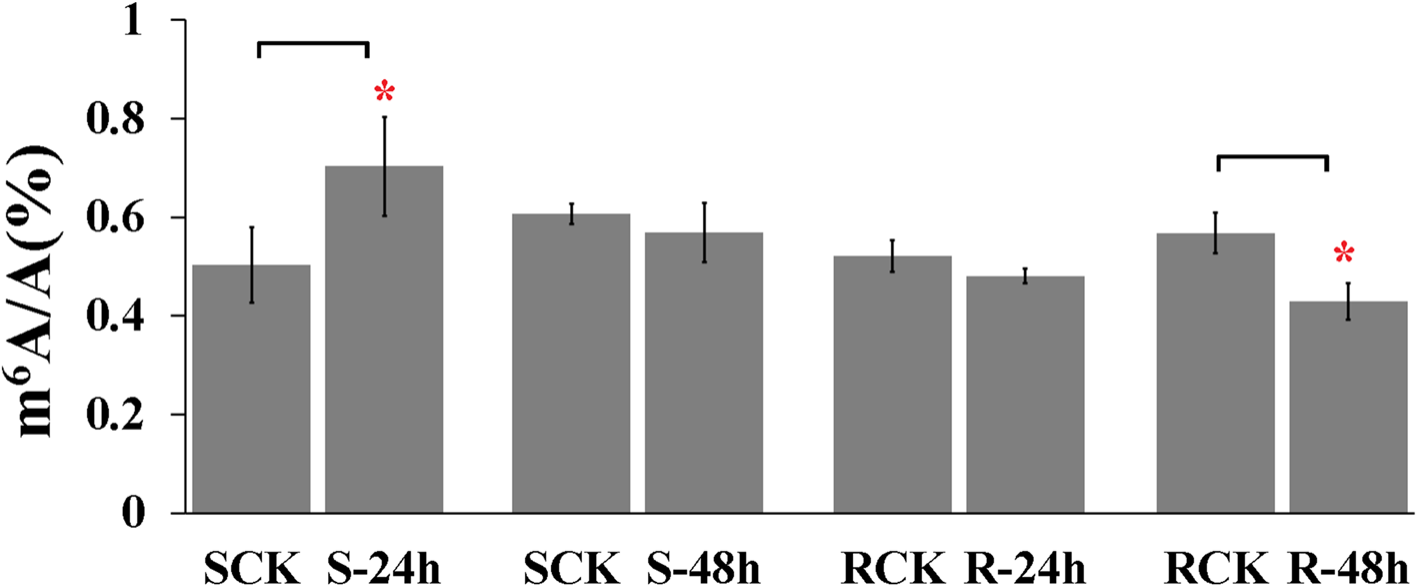
Extent of mRNA m^6^A modification levels in resistant and susceptible watermelon germplasms responsive to CGMMV infection. R1288 and S1252 were highly CGMMV-resistant and -susceptible watermelon germplasms, respectively. Watermelon leaves from the two germplasms were collected at 24 and 48 h after CGMMV infection. At same time, the un-infected mock samples were collected. Data are presented as the mean ± standard deviation (*n* = 3). Asterisks indicate significant differences (*P < 0.05 and **P < 0.01; Student’s *t*-test

To explore whether m^6^A modification responded to CGMMV infection, we assessed the mRNA m^6^A methylation levels in the control and CGMMV-infected leaves from resistant and susceptible watermelon (R1288 and S1252) by colorimetry (Fig. 1). The m^6^A level weakly decreased to 0.923-fold in resistant watermelon plants but significantly increased to 1.397-fold in susceptible watermelon plants 24 h after CGMMV infection. However, at 48 h, the m^6^A level in resistant watermelons significantly decreased to 0.757-fold, and the m^6^A level in susceptible watermelons weakly decreased compared to control samples. Therefore, CGMMV infection reduced leaf m^6^A levels of resistant watermelon.

### Transcriptome-wide mapping of m^6^A in watermelon

To investigate the transcriptome-wide map of m^6^A methylation in watermelon, a series of m^6^A- immunoprecipitation (IP) and matched input (non-IP control) libraries were constructed and sequenced (Supplementary file 5). This series included control and CGMMV-infected watermelon leaves, with two biological replicates each. High Pearson correlation coefficient (PCC) were determined for the abundance of confident peaks between biological replicates, representing highly reproducible results (Supplementary file Figure. S1). A total of 65.59-86.12 million reads were generated for each library, and after filtering out low-quality data, 65.11-85.41 million high-quality reads were mapped to watermelon reference genome (Charleston Gray). Furthermore, 63.03-82.59 million distinct reads were uniquely aligned to the genome and 22.85-30.35 million were mapped to splice reads (Table 1). Only m^6^A peaks consistently detected in both sample biological replicates were used for subsequent analyses.

**Table 1:** Sequenced and mapped reads in immunoprecipitation (IP) and the matched input (non-IP control) samples

Sample	Control_1 _input	Control_2 _input	CGMMV_2 _input	CGMMV_1 _input	Control_1	Control_2	CGMMV_2	CGMMV_1
Total reads	72.32	65.59	84.40	76.02	72.51	86.12	69.12	69.67
Total mapped reads	71.57	65.11	83.90	75.53	71.90	85.41	68.47	69.09
Multiple mapped	22.58	20.81	27.72	24.76	23.28	28.23	22.16	23.65
Uniquely mapped	69.31	63.03	81.13	73.05	69.57	82.59	66.26	66.72
Splice reads	25.47	23.91	30.35	26.97	24.29	29.07	23.21	22.85
Reads mapped in proper pairs	68.85	62.63	80.60	72.60	68.92	81.68	65.53	66.21

### m^6^A methylation is a common feature of watermelon mRNAs

**Fig. 2:**
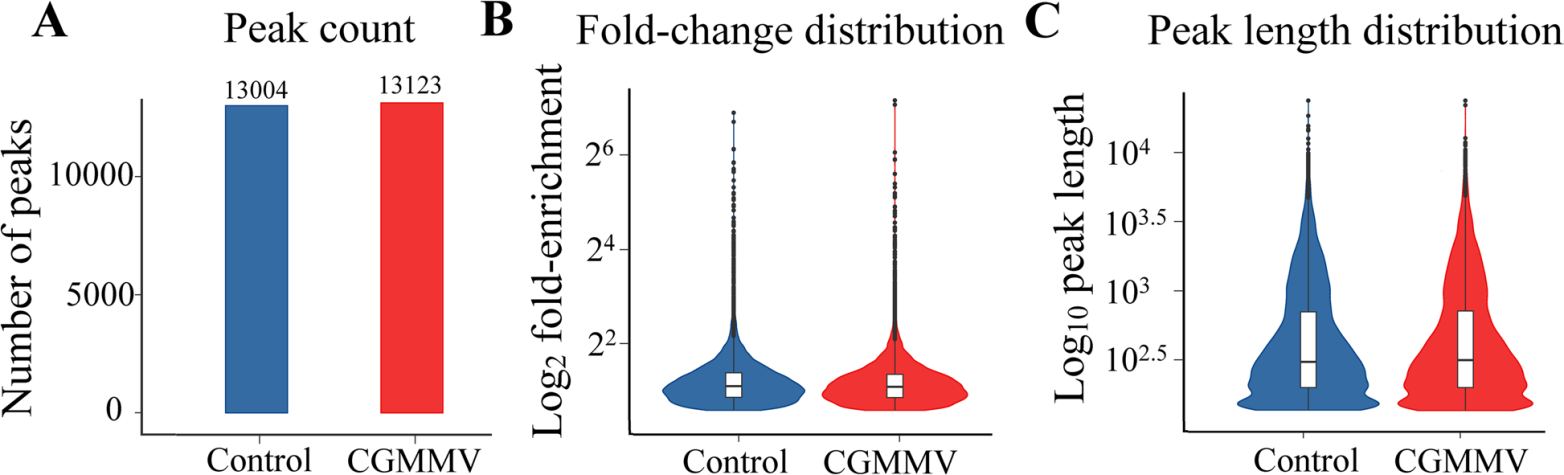
Characterization of m^6^A peaks in watermelon transcriptome. **A** Number of peaks in control and CGMMV infected samples. **B** Fold change distribution of m^6^A peaks in control and CGMMV infected samples. **C** Average length of peaks in control and CGMMV infected samples

At the genome level, 13,004 and 13,123 m ^6^A peaks with high confidence, distributed in 8,899 and 9,200 genes, were identified from the control and CGMMV-infected leaves, respectively, with an average of 1.45 m^6^A peaks within transcription units for each modified gene (Fig. 2A, Supplementary file 6). The enrichment multiple distribution analyses showed that more reads were enriched in the peaks of the CGMMV-infected watermelon than those of the control samples (Fig. 2B). The average length of the peaks was 671.38 and 689.34 bp in the control and CGMMV-infected samples, respectively (Fig. 2C).

**Fig. 3:**
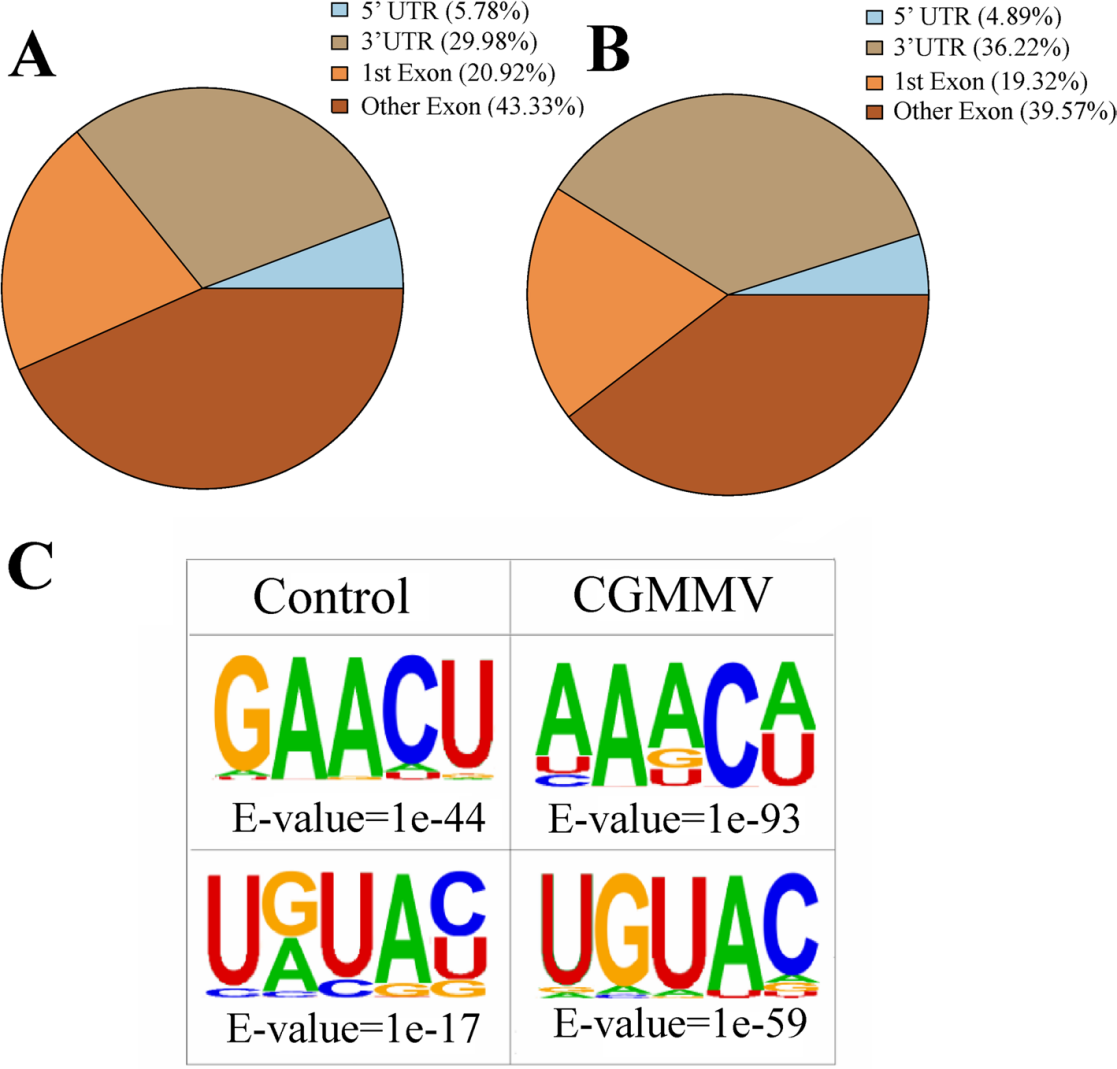
Characteristics of the m^6^A sites and sequence motifs in watermelon. **A** and **B** Pie charts depicting the fraction of m^6^A peak summits in four non-overlapping transcript segments from control and CGMMV infected samples. UTR, untranslated region; 1st Exon, the first exon. **C** The enriched RRACH and URUAY motifs identified from m^6^A peaks in watermelon with the HOMER online tool from control and CGMMV infected samples

The frequency of m^6^A modifications was unevenly distributed within RNA, being particularly highly enriched in mature mRNAs [20, 21]. The watermelon m^6^A peaks were highly abundant around the coding sequences (CDS) (58.89%-64.25%) and 3’-untranslated regions (3’-UTRs) (29.98%-36.22%) in all experimental samples (Fig. 3A and B). To identify enriched sequence motifs within watermelon m^6^A peaks, hypergeometric optimization of motifs enrichment were applied using the HOMER suite. The results indicated the most significant motif identified in watermelon m^6^A peaks was an RRACH motif (where R represents A/G, A is m^6^A, H represents A/C/U, and Y represents A, G, U, or C) with an E-value of 1e^-44^ and 1e^-93^ in control and CGMMV infected samples, respectively. The motif was found in 75.26% of all watermelon m^6^A peaks. Meanwhile, a URUAY motif was significantly enriched in 72.38% of watermelon m^6^A peaks (where Y represents C/U), with an E-value of 1e^-59^ and 1e^-17^ in control and CGMMV infected samples, respectively (Fig. 3C). These results suggest that m^6^A distribution and motifs in watermelon leaves are conserved, as reported in other species [11, 22, 23].

### m^6^A-modified genes in watermelon are involved in multiple signaling pathways and cellular processes

Gene ontology (GO) analysis indicated that watermelon m^6^A-modified transcripts were grouped into three categories: biological process, cellular component, and molecular_function (Supplementary file 8; Figure S2). Furthermore, these m^6^A-modified transcripts participated in multiple signaling pathways and cellular processes. The significantly enriched GO terms in biological process were involved in mRNA metabolism including mRNA processing (GO:0006397), rRNA processing (GO:0006364), mRNA splicing (GO:0000398, GO:0008380, and GO:0045292). For the cellular component, most of these modified genes were significantly enriched in the cytosol (GO:0005829), nucleolus (GO:0005730), and nucleus (GO:0005634) (Supplementary file 7; Figure S2). A The most enriched molecular functions were ATP binding (GO:0005524), RNA binding (GO:0003723), mRNA binding (GO:0003729), protein serine/threonine kinase activity (GO:0004674) and so on.

### Most watermelon differentially methylated peaks (DMPs) were hypo-methylated in response to CGMMV infection

MeTDiff was used to compare the abundance of m^6^A peaks between control and CGMMV-infected samples, and DMPs were defined based on a cutoff criterion of FPKM fold change ≥ 1.5 and P value < 0.05. Accordingly, 430 DMPs were identified, they were distributed among 422 differentially methylated genes (DMGs) (Supplementary file 8), and of these DMPs, 255 were hypo-methylated m^6^A peaks, whereas 175 were hyper-methylated m^6^A peaks. Moreover, 295, 115, and 21 DMPs were located in CDS, 3’-UTRs, and 5’-UTRs, respectively (Supplementary file 8; Figure S3).

**Fig. 4:**
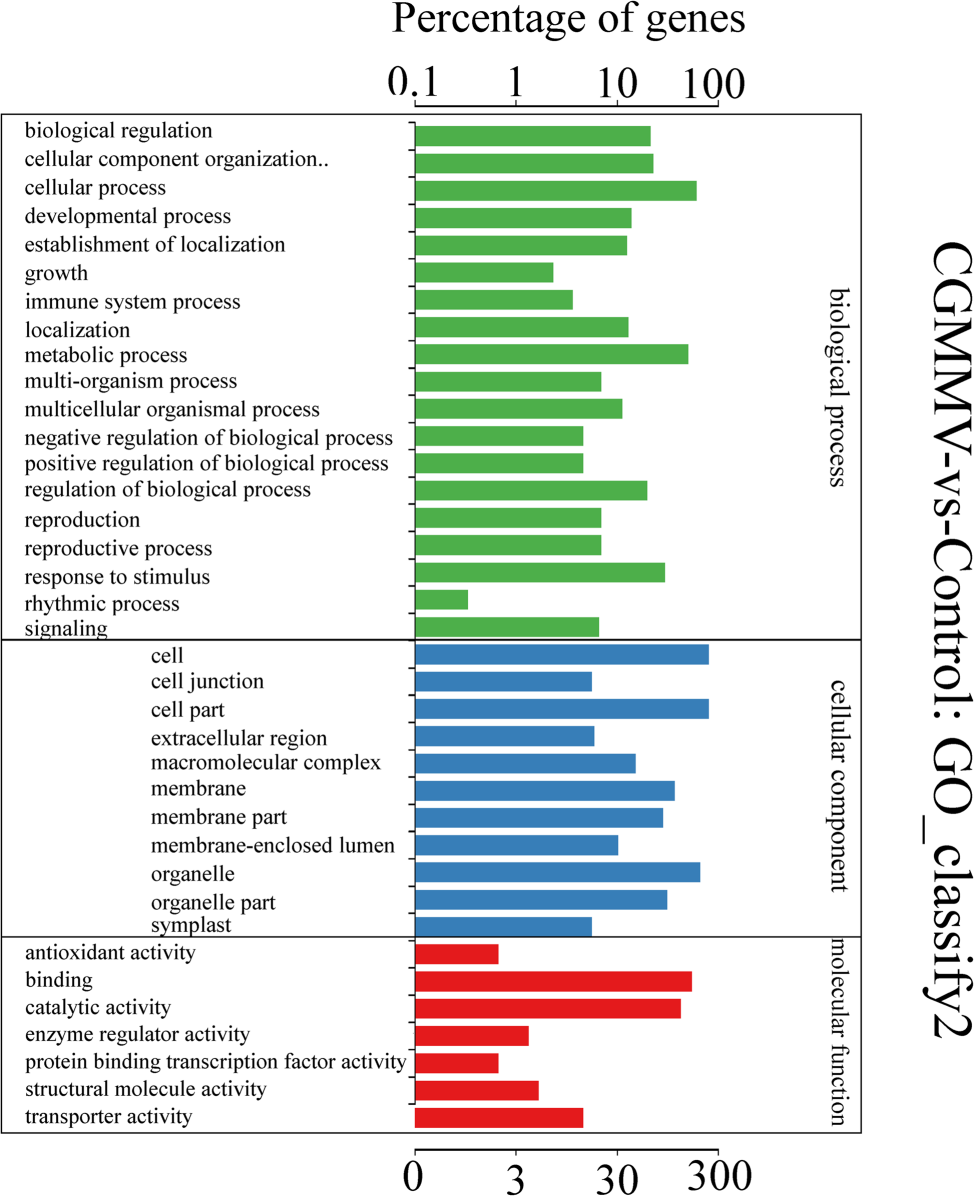
Gene ontology (GO) analysis of the biological process, cellular component, and molecular function annotations for the m^6^A-modified genes (DMGs) identified based on the m6A-seq data for control and CGMMV-infected samples. A term with *P* < 0.05 was considered to be significantly overrepresented

We performed GO analysis of the DMGs to explore their potential functional roles in response to CGMMV infection, and found that DMGs were involved in some important biological pathways such as cellular process, metabolic process, response to stimulus, cellular component organization or biogenesis, biological regulation, and so on (Fig. 4). Notably, 11 DMGs were involved in immune responses (Supplementary file 9-1), and were annotated as follows: zinc finger protein (ClCG01G014940), eukaryotic aspartyl protease family protein (ClCG05G026860), ankyrin repeat-containing protein (ClCG02G016860), 30 kDa cleavage and polyadenylation specificity factor subunit 4-like protein (ClCG03G006290), RNA-binding protein (ClCG09G021330), 26S protease regulatory subunit (ClCG00G004170), MLO-like protein (ClCG00G002750), CPR5 protein (ClCG01G019270), tetratricopeptide repeat protein (ClCG01G017250), ABC transporter family protein (ClCG07G013290), and late embryogenesis abundant (LEA) protein (ClCG10G018420). All of their m^6^A methylation levels were significantly downregulated from 0.042- to 0.666-fold except the genes encoding *MLO-like*, *CPR5*, and *LEA* genes (Supplementary file 9-1). Moreover, three DMGs (*ClCG03G016200*, *ClCG05G016300*, and *ClCG10G003630*), were annotated as DNA-directed RNA polymerase, RNA-dependent RNA polymerase, and DNA-directed RNA polymerase IV subunit 1 protein, respectively (Supplementary file 9-2). They all participated in the production of siRNA involved in RNA interference (GO:0030422) and m^6^A methylation levels increased from 1.528- to 2.042-fold after CGMMV infection (Supplementary file 9-2).

### Most watermelon differentially expressed genes (DEGs) were induced by CGMMV infection

**Fig. 5:**
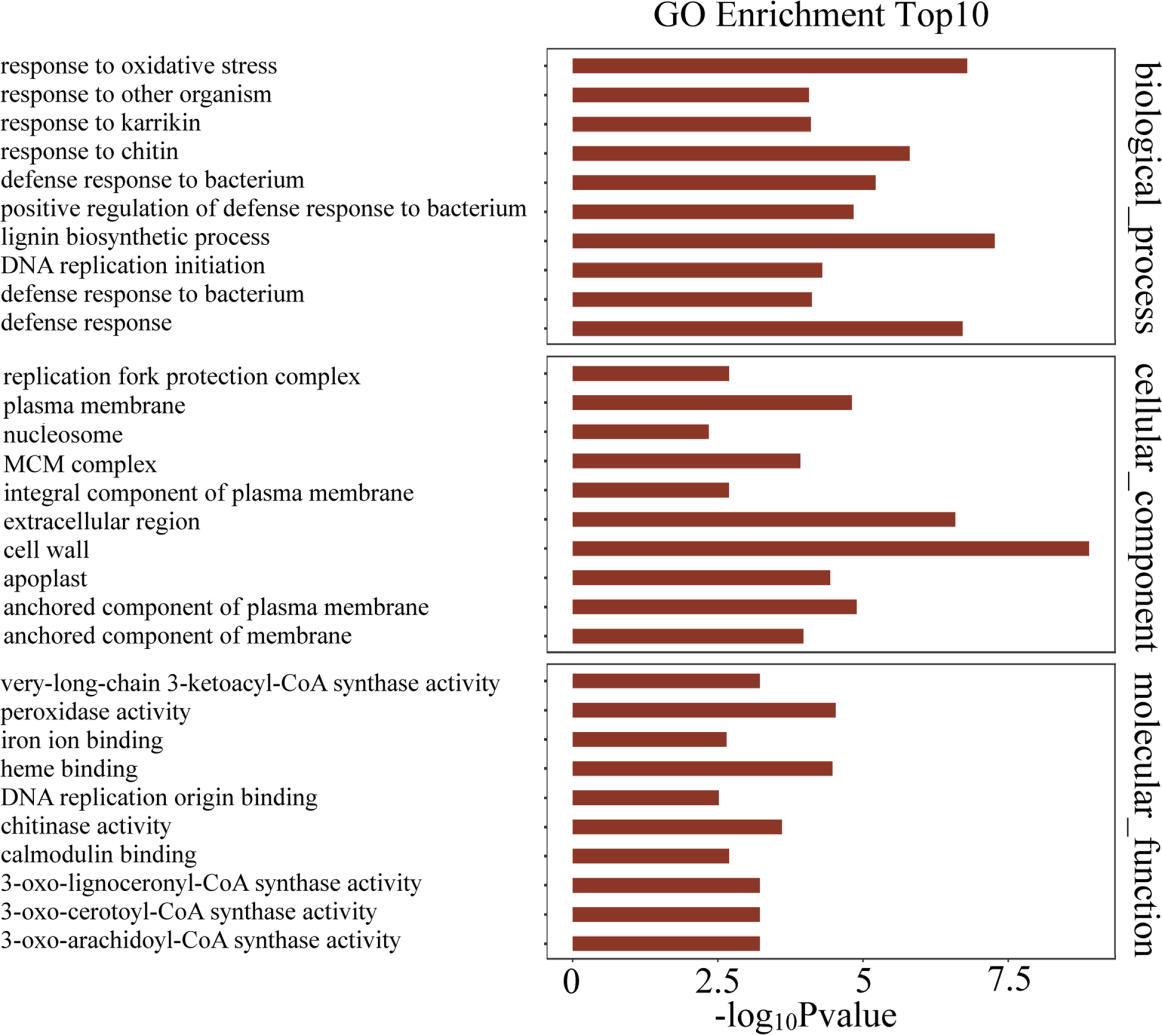
Gene ontology (GO) enrichment analysis (TOP10) of the biological process, cellular component, and molecular function annotations for the differentially expressed genes (DEGs) identified based on the RNA-seq data for control and CGMMV-infected samples. A term with *P* < 0.05 was considered to be significantly overrepresented

We performed RNA-seq analysis to quantify the expression levels of m^6^A elements in resistant watermelon leaves in response to CGMMV infection. Genes with fold change ≥ 1.5 and P value < 0.05 between libraries were considered to be significantly differentially expressed genes (DEGs). A total of 1,855 DEGs were identified, of which 1,230 were upregulated and 625 were downregulated 48 h after CGMMV infection (Supplementary file 10). Among these DEGs, 45.88% contained m^6^A peaks, which was significantly higher than the rate for all of watermelon transcripts (39.43% - 40.76%). GO enrichment analysis (top 10 enrichment scores) indicated that these DEGs were significantly enriched in various biological process pathways including defense response (GO:0006952), response to oxidative stress (GO:0006979), defense response to bacterium (GO:1900426 and GO:0042742), response to chitin (GO:0010200), DNA replication initiation (GO:0006270), and so on. In molecular_function, the DEGs were significantly enriched in pathways related to heme binding (GO:0020037), iron ion binding (GO:0005506), calmodulin binding (GO:0005516), peroxidase activity (GO:0004601), and chitinase activity (GO:0004568). Besides, they highly located in plasma membrane (GO:0005886) (Fig. 5).

### m^6^A machinery components involved in regulation of m^6^A modification in virus-infected watermelon plants

**Fig. 6:**
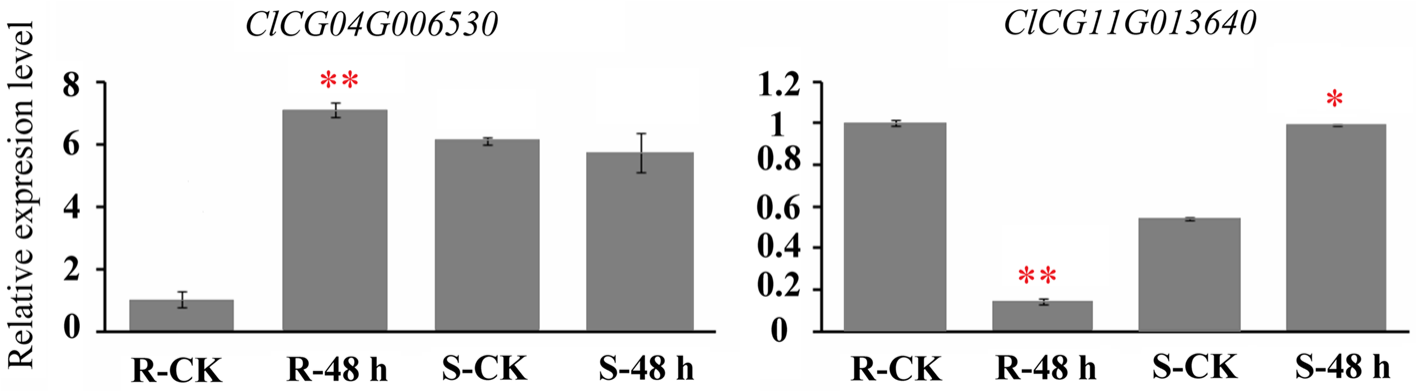
The response patterns of two m^6^A demethylases (ALKBHs) in resistant and susceptible watermelon germplasms to CGMMV infection. For other details, see Fig. 1

All watermelon m^6^A machinery components including three m^6^A methylases (*ClMTA*, *ClMTB*, and *ClMTC*), twelve demethylases (*ClALKBH1*-*ClALKBH12*), and five m^6^A readers (*ClECT1*- *ClECT5*), were identified from watermelon genome (Supplementary file 11). None of these m^6^A gene expressions showed statistically significant differences in response to CGMMV infection via RNA-seq analysis, except two m^6^A demethylases, *ClALKBH4B* (*ClCG04G006530*) and *ClALKBH11B* (*ClCG11G013640*). After infection, the expression level of *ClALKBH11B* dropped to 0.493-fold, but that of *ClALKBH4B* expression increased to 1.519-fold. Meanwhile, the expression patterns of these two genes were also verified by qRT-PCR in resistant and susceptible watermelon varieties under CGMMV infection. Similarly, *ClALKBH4B* expression increased 7.086-fold in resistant varieties, but did not change (0.931-fold) in susceptible varieties 48 h after CGMMV infection. *ClALKBH11B* expression decreased to 0.142-fold in resistant watermelon, but increased to 1.828-fold in susceptible watermelon (Fig. 6). Moreover, a m^6^A reader, *ClECT1* (*ClCG03G006290*), exhibited significantly decreased m^6^A (0.666-fold) under CGMMV infection in m^6^A-seq (Supplementary file 9).

### Conjoint analysis of DMGs and DEGs responsive to CGMMV infection

To explore a potential correlation between transcript levels and m^6^A mRNA methylation in response to CGMMV infection, a conjoint analysis of DMGs and DEGs was conducted and 59 differentially methylated and expressed genes (DMEGs) were identified. The DMEGs were mainly divided into four groups, including 22 hypomethylated but upregulated genes (hypo-up) (37.29%), 16 hypermethylated as well as upregulated genes (hyper-up) (27.12%), 17 hypomethylated as well as downregulated genes (hypo-down) (28.81%), and 6 hypermethylated but downregulated genes (hyper-down) (10.17%) (Supplementary file 12). Functional annotation analyses indicated that these genes were involved in multiple roles and signaling pathways such as resistance response, secondary biosynthesis and metabolism, and RNA processes. Among them, six transcript factors (TFs) including *WRKY DNA-binding protein 29 (WRKY29)* (*ClCG07G007870*), *ethylene-responsive transcription factor (ERF)* (*ClCG10G017140*), *MYB domain protein 6*8 (MYB68) (*ClCG08G016830*), *heat stress transcription factor (HSF)* (*ClCG01G016810*), *GATA transcription factor* (*ClCG07G008920*), and *zinc finger (bZIP)* (*ClCG08G016400*) were identified. Except for *WRKY29*, all of their m^6^A methylation levels decreased from 0.55- to 0.65-fold, but transcript patterns displayed diverse responses. The transcript levels of *WRKY29*, *HSF*, and *bZIP* were upregulated 1.85- to 3.39-fold, but *MYB68*, *ERF*, and *GATA* were downregulated from 0.14- to 0.57-fold. Seven genes involved in sugar metabolism and signaling encoding were identified , including alpha-l-fucosidase (*ClCG01G001290*), glycosyltransferase family protein (*ClCG01G025940*), C2 Calcium/lipid-binding plant phosphoribosyl transferase family protein (*ClCG04G005410*), O-fucosyltransferase family protein (*ClCG10G021720*), glutamine-fructose-6-phosphate amino transferase (*ClCG11G009710*), UDP-D-GLUCURONATE 4-EPIMERASE 3 family protein (*ClCG10G003090*), and endo-1,4-beta-glucanase (*ClCG11G011070*). Among these seven genes, the m^6^A modification levels of four genes including *ClCG01G001290*, *ClCG04G005410*, *ClCG01G025940*, and *ClCG10G003090* were significantly downregulated from 0.40- to 0.60-fold, while the other three genes were upregulated from 1.55- to 2.32-fold. Five plant resistance genes were identified, including encoded receptor lectin kinase-like protein (ClCG10G022400), late embryogenesis abundant (LEA) hydroxyproline-rich glycoprotein protein (ClCG10G018420), ankyrin repeat-containing proteins (ClCG02G016860 and ClCG01G009830), and ABC transporter family pleiotropic drug resistance protein (ClCG07G013290). Among them, the m^6^A methylation levels of *ClCG10G018420* and *ClCG02G003390* significantly increased but the other three genes were decreased. Meanwhile, except for *ClCG01G009830*, all the four gene expressions were induced. Five genes participated in genetic information processing, among them, *ClCG09G005490* and *ClCG08G009480* were involved in transcription, with increased m^6^A modification and decreased transcript levels, And *ClCG01G025960* involved in replication and repair, showed increased m^6^A modification and transcript levels (Supplementary file 12).

### Validation of m^6^A methylation and transcript levels of DEGs

**Fig. 7:**
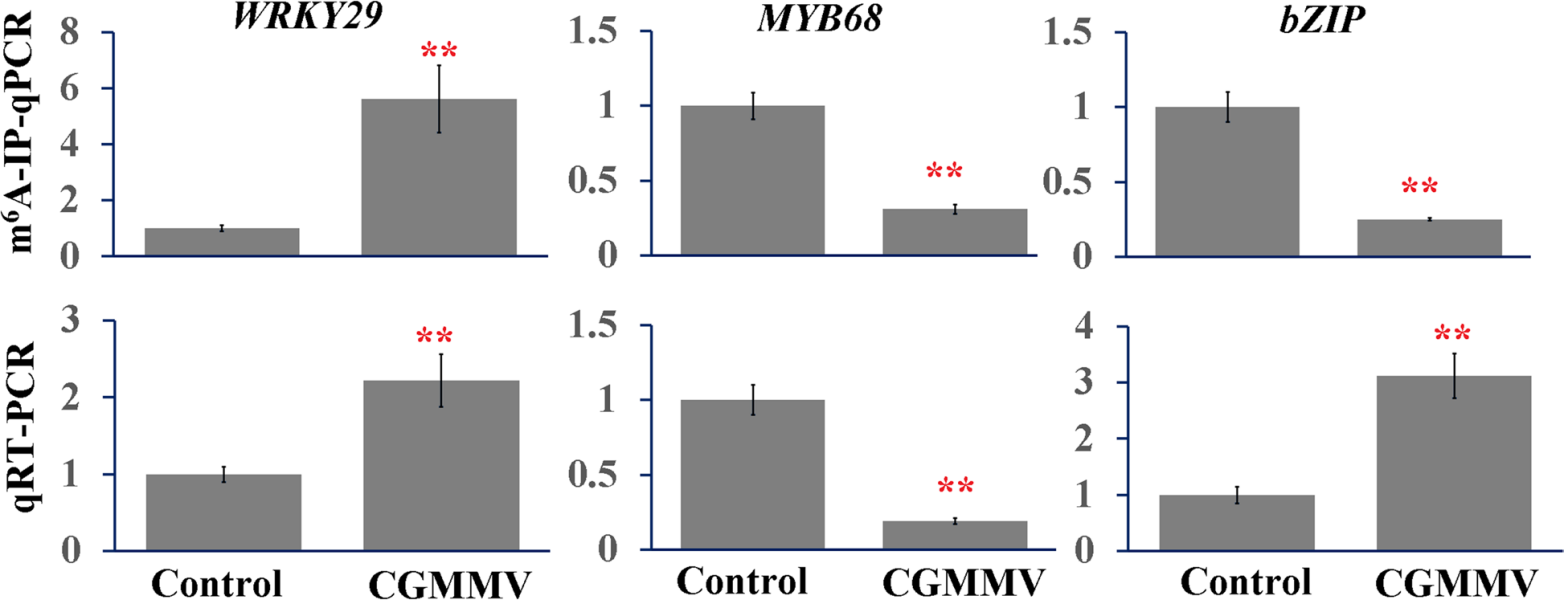
Validation of m^6^A peaks in three watermelon CGMMV-responsive genes using quantitative RT-PCR (qRT-PCR) and m^6^A-immunoprecipitation(IP)-qPCR (m^6^A-IP-qPCR)

We confirmed the m^6^A methylation and transcript levels of *WRKY29*, *MYB68*, and *bZIP* in watermelon by m^6^A-IP-qPCR and qRT-PCR. As expected, methylation levels of *MYB68* and *bZIP* genes were downregulated 0.19- and 0.25-fold, respectively, whereas the methylation level of *WRKY29* was upregulated 2.22-fold. Meanwhile, the expression levels of *WRKY29* and *bZIP* were upregulated 5.62- and 3.12-fold (Fig. 7), but *MYB68* expression was repressed (0.31-fold). These results confirm that our m^6^A-seq and RNA-seq data were accurate and robust.

## Discussion

Plants have developed complex molecular mechanisms to resist viruses. Some RNA epigenetic modifications involved in the post-transcriptional regulation of virus-induced genes play significant roles in plant antiviral processes. As one of the most important RNA methylations in eukaryotes, m^6^A methylation is important for distinct RNA processing steps [24] and was recently identified regulating virus infection in plants [13, 14]. However, presently there is no transcriptome-wide study on the m^6^A modification in early response to virus infection in plants. CGMMV, a species of the *Tobamovirus genus*, has caused considerable yield losses in commercial watermelon production worldwide. To determine the response patterns of RNA m^6^A modification to CGMMV infection in watermelon, libraries were constructed from watermelon leaves collected 48 h after CGMMV to identify the responsive genes at the m^6^A methylation level using high-throughput sequencing technology (m^6^A-seq).

### The m^6^A modification features in watermelon

Our study is the first to generate and globally characterize the transcriptome-wide RNA m^6^A modification profiles in watermelon. For the watermelon genome, out of all transcripts, 8,899 to 9,200 genes (39.43-40.76%) were identified as m^6^A modified genes (Supplementary file 6). The proportion of modified genes in watermelon was higher than that in Arabidopsis (19.58-22.74%) and tomato (26.48-27.71%) [22, 23]. Additionally, over 30% of m^6^A-modified watermelon genes had more than one peak, which was obviously higher than the rate in tomato (8.27%) and maize (5.88%). To date, two conserved motifs, RRACH and URUAY, have been identified in plants. Specifically, URUAY is a plant-specific consensus motif enriched within m^6^A peaks generated for Arabidopsis, tomato, rice, and maize [11, 22, 23, 25]. In our study, m^6^A peaks in watermelon also contained the motifs of RRACH and URUAY, with frequency of 75.26 and 72.38%, respectively. Further studies indicate that the enrichment *E*-values of the RRACH motif were clearly lower than those of the URUAY motif in watermelon and Arabidopsis [22] (Fig. 3C), but the opposite result was observed in maize. The different m^6^A site biases between monocot (maize) and dicot (Arabidopsis and watermelon) plants is probably related to their unique differences in structure and function of m^6^A methylation. Almost all watermelon m^6^A peaks (94.22-95.11%) were located in 3’-UTRs and CDS (Fig. 3A and B), which is similar to the results in Arabidopsis, tomato, rice, and maize [11, 22, 23]. GO analysis indicated that m^6^A modified-genes in watermelon were significantly enriched in multiple signaling pathways and cellular processes, implying their multiple roles (Fig. 4, Supplementary file 7). Collectively, m^6^A is a common feature in a substantial fraction of watermelon mRNA. m^6^A modification distributions and characteristics were highly conserved in watermelon, but more abundant than in other reported plants.

### Potential roles of m^6^A modification in early response to CGMMV in watermelon

Studies have found that the m^6^A methylation level of plant hosts is reprogrammed in response to viral infection [13, 14]. We found the global m^6^A level was regulated by CGMMV infection (Fig. 1), it was more significantly downregulated in resistant, than susceptible, watermelons. This implies that a global m^6^A-hypomethylation probably activates watermelon defense responses at the early stage of CGMMV infection. Furthermore, 422 DMGs responsive to CGMMV infection were screened out by m^6^A-seq, of which 60.42% were the hypomethylated after the CGMMV infection. These results suggest that m^6^A modifications might negatively affect watermelon responses to CGMMV even further. Meanwhile, 1,855 DEGs responsive to CGMMV infection were identified in watermelon via RNA-seq, of which 66.31% were induced. This is consistent with previous findings that m^6^A methylation is generally negatively correlated with transcript abundance [23].

The m^6^A methylation is directly controlled by m^6^A methylases, demethylases, and binding (reader) proteins. In watermelon, we found a m^6^A reader, homologous with Arabidopsis *ECT1/CPSF30*, was hypomethylated by m^6^A modification after CGMMV infection (Supplementary file 9). In Arabidopsis, *ECT1/CPSF30* played important roles in immune responses by regulating the splicing of the 3’ end of mRNA [26]. In tobacco, the m^6^A global level was decreased because the expression of some *ALKBHs* were increased after TMV infection [14]. In watermelon, the transcript levels of two m^6^A demethylases, *ClALKBH4B* and *ClALKBH11B*, were significantly changed in RNA-seq and qRT-PCR data under CGMMV infection (Fig. 6, Supplementary file 11). *ClALKBH4B* expression was significantly induced by CGMMV infection in resistant watermelon plants, but seemed to be unaffected in susceptible plants. Therefore, we suspected that the decreased expression of *ClALKBH4B* resulted in the hypomethylation of most DMGs in m^6^A-seq.

Virus-induced gene silencing, as one type of posttranscriptional gene silencing (PTGS), is a common antiviral strategy used by plants. Virus infection is initiated through the transcription catalyzed by an RNA-dependent RNA polymerase to produce double-stranded RNA (dsRNA) [27, 28]. The dsRNA is cleaved to generate small interfering RNA (siRNA), which is recruited by host RNA-induced silencing complexes to repress viral gene transcription and translation. Arabidopsis AtALKB9B was found to colocalize with siRNA-body and is implicated in PTGS through the synthesis of dsRNAs to generate siRNAs (Martínez-Pérez et al. 2017). We found three DMGs including *ClCG03G016200*, *ClCG05G016300*, and *ClCG10G003630*, participated in the production of siRNA and all of them were m^6^A-hypermethylated after CGMMV infection (Supplementary file 9). This suggests that m^6^A methylation in watermelon was probably involved in virus-induced gene silencing to resist CGMMV infection.

In terms of plant-virus interactions, plants have evolved immune systems that recognize the presence of viruses and initiate the expression of effective defense response factors. Recently, two studies have reported on transcriptome and proteomic profiles of CGMMV-infected and mock-inoculated watermelon leaves or fruit [19, 29]. We compared them with our RNA-seq data and found that the number of DEGs identified in previous RNA-seq data (1641 DEGs) was similar to our study (1855 DEGs). Most of the DEGs or differentially accumulated proteins (DAPs) in the three studies were induced by CGMMV infection and functional analyses indicated they enriched in the pathways involved in stress response, photosynthesis, secondary metabolism, and so on [19, 29]. These results further validated the common pathways in watermelon involved in CGMMV response. To further explore the relationship between the m^6^A methylation extent and the transcript level in response to CGMMV infection, we identified 59 genes as DMEGs and they were important candidate CGMMV-responsive genes at transcriptional and post-transcriptional (m^6^A modification) levels. Most of their homologous genes have been found to participated in plant innate immunity. For example, six TFs were identified and all of their m^6^A methylation levels decreased except *WRKY29*, and *WRKY29*, *HSF*, and *bZIP* transcript levels increased in response to CGMMV infection (Supplementary file 12). These TFs have been proven to participate in plant-pathogen interaction pathways. *WRKY* TFs were involved in the defense signaling pathway associated with *Tomato yellow leaf curly virus* infection [30]. Arabidopsis *HSF* was involved in the regulation of *Pdf1.2* expression and pathogen resistance [31]. And *bZIP* TFs were important for plant innate immunity as they regulated the expression of genes associated with PAMP- and, effector-triggered immunity, and hormone signaling networks [32, 33].

Plant antiviral mechanisms result in the regulation of plant carbohydrate allocation and signaling. Soluble sugars and starch accumulate in the leaves to initiate viral replication, resulting in decreased photosynthesis and increased respiration [34, 35]. We identified seven DMEGs involved in sugar metabolism and signaling (Supplementary file 12). Most of them were m^6^A-hypomethylated but the transcript levels were upregulated after CGMMV infection. Most of their homologous genes were found to have participated in plant innate immunity. Notably, we compared these DMEG with the previous RNA-seq results and found two DMEGs (*ClCG11G011070* and *ClCG10G021720*), both involved in suger metabolism and signalling, were also significantly induced by CGMMV infection in the previous RNA-seq data [29]. These m^6^A-regulated genes probably affect soluble sugar and starch accumulation to participate in CGMMV response.

It's worth mentioning that, only two genes (*ClCG02G022850* and *ClCG09G016700*), both encoded ABC transporters, out of all DMEGs contained two DMPs, and their transcript levels were induced under CGMMV infection, while the two m^6^A peaks in *ClCG02G022850* were significantly hypermethylated, but only one peak in *ClCG09G016700* were hypermethylated in response to CGMMV infection (Supplementary file 12). ABC transporter proteins have been found to play important roles in resisting cell wall penetration and haustorium formation, and improving the resistance to some fungal and oomycete pathogens [36]. These studies suggested that the m^6^A modified ABC transporter in watermelon probably play important roles in CGMMV responses via a complex regulatory mechanism.

## Conclusions

m^6^A modification is essential for plant growth and development and stress responses, including plant antiviral immunity. In this study, we performed m^6^A-seq to analyze transcriptome-wide m^6^A methylation profile of watermelon leaves under CGMMV infection. And the results indicated the distributions and motifs of m^6^A methylation were highly conserved while the modification rates was obviously higher in watermelon than that in other reported plants. In further, we proposed a preliminary hypothesis to explain the mechanism of watermelon resistance to viral infection via the m^6^A modification; m^6^A demethylase gene *ClALKBH4B* could be significantly induced in early response to CGMMV in resistant watermelon, and the decreased expression of *ClALKBH4B* resulted in the methylation of numerous target modified-genes were downregulated. The m^6^A methylation of the transcripts was generally negatively correlated with transcriptional level as reported previously [23], so some downstream defense response factors involved in virus-induced gene silencing, TFs, plant carbohydrate allocation and signaling, and so on, were induced at transcript level via RNA-seq analysis and a series of plant immune responses were activated at the early stage of CGMMV infection. This study firstly provide insights into the m^6^A modification features in watermelon leaf and reveal the roles and regulatory mechanisms of m^6^A modification under CGMMV infection in watermelon.

## Methods

### Plant materials and CGMMV inoculation

We used two watermelon germplasms, namely, R1288 (a highly CGMMV-resistant watermelon germplasm, R) and S1252 (a highly CGMMV-susceptible watermelon germplasm, S), that were identified through resistance screening (Supplementary file Fig. S4). Watermelon seedlings were grown under standard culture conditions (25 °C and a 16-h light/8-h dark cycle) in a greenhouse. For CGMMV inoculation, seedlings at the two true-leaf stage of the two watermelon germplasms were agroinfiltrated with Agrobacterium carrying CGMMV infectious clone, as described previously [29]. Meanwhile, the seedlings of the two watermelon germplasms were inoculated with sodium phosphate buffer (pH 7.2) without virus and were used as control (Mock). At 24 h post-inoculation, at least three leaves each from un-infected control and infected plants were collected and mixed to a biological replicate and three biological replicates were obtained for watermelon germplasm R1288 and S1252, respectively. Similarly, samples were collected at 48 h post-inoculation. Samples collected from the un-infected and infected plants of R1288 and S1252 at 24 and 48 h post-inoculation, respectively, were used for global m^6^A methylation quantification and quantitative RT-PCR (qRT-PCR) analyses. Samples collected from from the un-infected and infected R1288 plants at 48 h post-inoculation were used for high-throughput m^6^A-seq and RNA-seq.

### Global mRNA m^6^A methylation assay

Global m^6^A methylation assay was performed as reported [23]. Firstly, total RNA was extracted from harvested leaves by using RNAiso Plus (TaKaRa). And PolyA+mRNA selection was isolated using a Dyna beads mRNA Purification Kit (Life Technologies). Then we measured the global m^6^A levels in transcripts with an EpiQuik m^6^A RNA Methylation Quantification Kit (Colorimetric) (Epigentek) according to the manufacturer’s protocol.

### Identification of watermelon m^6^A elements

The hidden Markov model (HMM) profile for the MT70 superfamily (PF05063), ALKBH superfamily (PF13532, clavaminate synthase-like domain), and YTH domain-containing protein (PF04146) sequences were downloaded from the PFAM website (http://pfam.xfam.org/), and the HMMER tool was applied to identify orthologous proteins in the watermelon reference genome (*Charleston Gray*) (http://cucurbitgenomics.org/). These m^6^A genes were named according to their counterparts in Arabidopsis.

### High-throughput m^6^A-seq and RNA-seq

Resistant watermelon leaves (R1288) were collected at 48 h post-CGMMV inoculation and mock and used for the m^6^A-seq, which was carried out as reported [20]. Specifically, total RNA was extracted and fragmented into approximately 200 nucleotide-long fragments. These fragments were incubated and purified as previous reported [15]. m^6^A-seq was performed on the Illumina HiSeq platform at Shanghai OE Biotech Co., Ltd.

### Sequencing data analysis

The raw reads were trimmed to remove adapters and low-quality bases with the Trimmomatic 0.36 [37], and their quality was assessed with the FastQC program (https://www.bioinformatics. babraham.ac.uk/projects/fastqc). The reads were aligned to the watermelon reference genome (Charleston Gray) with HISAT2 and annotated. All the uniquely mapped reads with a MAPQ ≥ 13 were retained for follow-up analyses [20].

Chipseeker software [38] was used for identifying m^6^A peaks in each anti-m^6^A IP sample, with the corresponding input sample serving as a control. A stringent cutoff threshold for the false discovery rate (FDR) < 0.05 was used to obtain high-confidence peaks. Only peaks consistently called in both two independent biological samples were considered as high-confidence peaks and used for subsequent analyses, and these peaks were annotated with PeakAnnotator (version 2.0) [39].

Differentially methylated peaks between samples were determined with MeTDiff software [40] using the following criteria: FoldChange ≥ 1.5 and P value < 0.05. The m^6^A-enriched motifs were identified with HOMER (version 4.7; http://homer.ucsd.edu/homer/) [41]. All peaks mapped to mRNAs were used as target sequences, and exon sequences except for the peak-containing sequences were used as the background sequences. The motif length was restricted to six nucleotides and the E-value threshold was 1E-5. Visualization analysis of m^6^A peaks was conducted using Integrated Genome Browser (IGB, version 9.0.2) [42]. Gene Ontology (GO) analysis of m^6^A-modified genes was performed with the Gene Ontology Consortium (http://www.geneontology.org/). GO terms with a Bonferroni corrected P value < 0.05 were considered to be significantly enriched for individual genes.

RNA-seq reads were aligned to the watermelon (*Charleston Gray*) reference genome, with default settings except for the maximum intron length, which was set to 10 kilobase (kb). Unique mapped reads were used as the input for Cufflinks v2.2.1 [43], which was used to normalize and estimate gene expression levels in terms of fragments per kilobase of transcript per million mapped reads (FPKM = counts of mapped fragments×10^9^/ [transcript length×total count of the mapped fragments]). Differentially expressed genes (DEGs) were analyzed using the Cuffdiff program of Cufflinks [43]. The watermelon (*Charleston Gray*) reference genome sequences and annotations were downloaded from the CuGenDB database (http://cucurbitgenomics.org/).

### Validation of transcriptome data by m^6^A-IP-qPCR and qRT-PCR

To validate m^6^A methylation and expression levels revealed by the transcriptome data, watermelon leaves were collected at 48-h post-CGMMV inoculation and mock as described above and used for RNA extraction. The m^6^A-IP-qPCR was performed as previously described, with some modifications [23, 44]. The purified mRNA was fragmented into approximately 200 nucleotide sequences, and then stopped, and the mRNA fragments were purified by ethanol precipitation and resuspended. The fragmented mRNA was then resuspended and 100 μL of fragmented mRNAs used as the input sample (input mRNA). Another 100 μL of fragmented mRNAs were incubated with an anti-m^6^A polyclonal antibody. After washing twice with high-salt buffers and twice with IP buffers, the bound mRNAs were eluted from the beads, and immune-precipitated mRNA fragments were resuspended in 5 μL DEPC-treated water. Then, the immunoprecipitated mRNA and pre-immunoprecipitated mRNA (input mRNA) were reverse transcribed with random hexamers using M-MLV reverse transcriptase (Takara) to generate the cDNA for the IP and pre-IP samples. The cDNA was used as the template for the m^6^A-IP-qPCR and qRT-PCR. The enrichment of m^6^A methylation in specific gene regions was determined using the cycle threshold (CT) 2^(-ΔCT)^ method [45]. The *β-actin* gene was used as an internal control [46]. The primers used for m^6^A-IP-qPCR and qRT-PCR are listed in Supplementary file 13.

## Supplementary Information

**Additional file 1: Supplementary file 1 Figure S1.** High Pearson correlation coefficient (PCC) for the abundance of confident peaks between biological replicates.

**Additional file 2: Supplementary file 2 Figure S2.** Gene ontology (GO) analysis (GO: level 2) of the m^6^A-containing transcripts identified in m^6^A -seq in watermelon.

**Additional file 3: Supplementary file 3 Figure S3.** Pie charts depicting the fraction of DMPs in four non-overlapping transcript segments.

**Additional file 4: Supplementary file 4 Figure S4.** The phenotype of two watermelon germplasm (R1288 and S1252) plants infected CGMMV 20 d.

**Additional file 5: Supplementary file 5.** Summary of m^6^A -seq information for CGMMV-infected and control samples.

**Additional file 6: Supplementary file 6.** High-confidence m^6^A peaks identified for control samples and CGMMV-infected samples.

**Additional file 7: Supplementary file 7.** Gene ontology (GO) analysis of the biological process, molecular function, and cellular component for the m^6^A -containing transcripts identified in m^6^A -seq in watermelon.

**Additional file 8: Supplementary file 8.** Characteristics of differentially methylated peaks (DMPs) in CGMMV-infected and control samples.

**Additional file 9: Supplementary file 9.** The m^6^A modification level of DEGs involved in immune responses and the production of siRNA involved in RNA interference in this study.

**Additional file 10: Supplementary file 10.** DEGs identified in RNA-seq in this study.

**Additional file 11: Supplementary file 11.** The response patterns of watermelon m^6^A elements to CGMMV infection in RNA-seq data.

**Additional file 12: Supplementary file 12.** The differentially m^6^A methylated and expressed genes (DMEGs) identified in this study.

**Additional file 13: Supplementary file 13.** Primers used for m^6^A-immunoprecipitation(IP)-qPCR (m^6^A -IP-qPCR) and Real-time quantitative PCR (qPCR).

## Data Availability

The raw sequencing data and processed peaks data for m^6^A-seq have been uploaded to the NCBI database under the SRA accession number: PRJNA597500 (https://www.ncbi.nlm.nih.gov/ bioproject/PRJNA597500). All the public databases used in this study are open. All the raw data generated in this study are included in the article and the supplemental files.

## Abbreviations

m^6^A: N6-methyladenosine
CGMMV: *Cucumber green mottle mosaic virus*
WYMV: *Wheat yellow mosaic virus*
RBSDV: *Rice black-streaked dwarf virus*
RSV: *Rice stripe virus*
AMV: Alfalfa mosaic virus
TMV: *Tobacco mosaic virus*
DMGs: Differentially methylated genes
GO: Gene Ontology
ALKBHs: Alkylated DNA repair proteins AlkB homologs
ECT: EVOLUTIONARILY CONSERVED C-TERMINAL REGION
CPSF30: Cleavage and Polyadenylation Specificity Factor 30
AMV: *Alfalfa mosaic virus*
m6A-IP-qPCR: m^6^A-immunoprecipitation (IP)-qPCR
R: Resistant varieties
S: Susceptible varieties
HMM: Hidden Markov model
FDR: False discovery rate
DEGs: Differentially expressed genes
qRT-PCR: Quantitative RT-PCR
IP: Immunoprecipitation
PCC: Pearson correlation coefficient
CDS: Coding sequences
UTRs: DMEGs: Untranslated regions: differentially methylated and expressed genes
LEA: Late embryogenesis abundant
PTGS: Posttranscriptional gene silencing
dsRNA: Double-stranded RNA
siRNA: Small interfering RNA
