# Supplementary figures and images for: Transcriptome-wide N6-methyladenosine (m6A) methylation in watermelon under CGMMV infection
Source: BMC Plant Biol. 2021 Nov 8;21:516. doi: 10.1186/s12870-021-03289-8 (PMC8574010; doi:10.1186/s12870-021-03289-8)

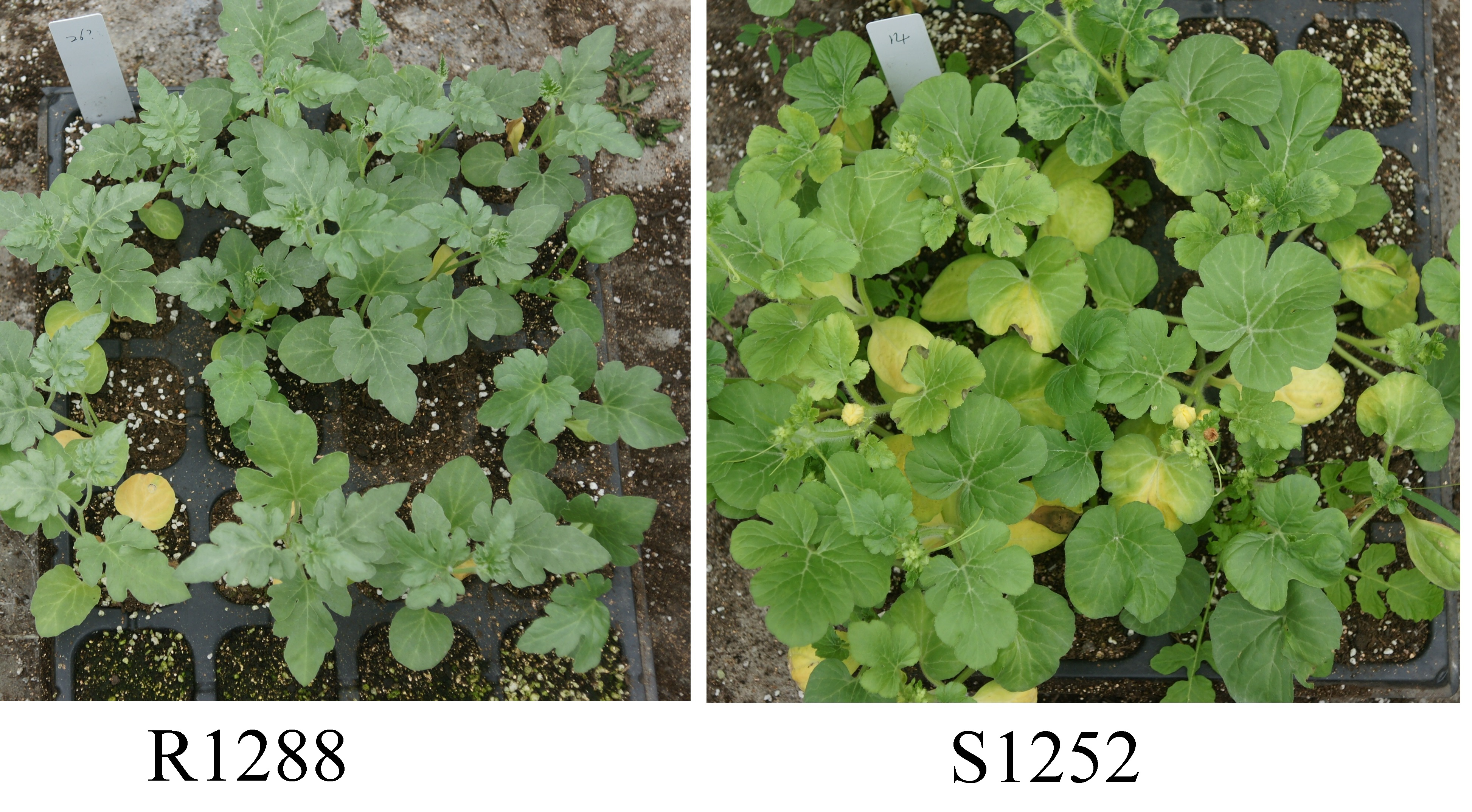

Supplement: Supplementary file 1 — Additional file 1: Supplementary file 1 Figure S1. High Pearson correlation coefficient (PCC) for the abundance of confident peaks between biological replicates. [file 12870_2021_3289_MOESM1_ESM.png]

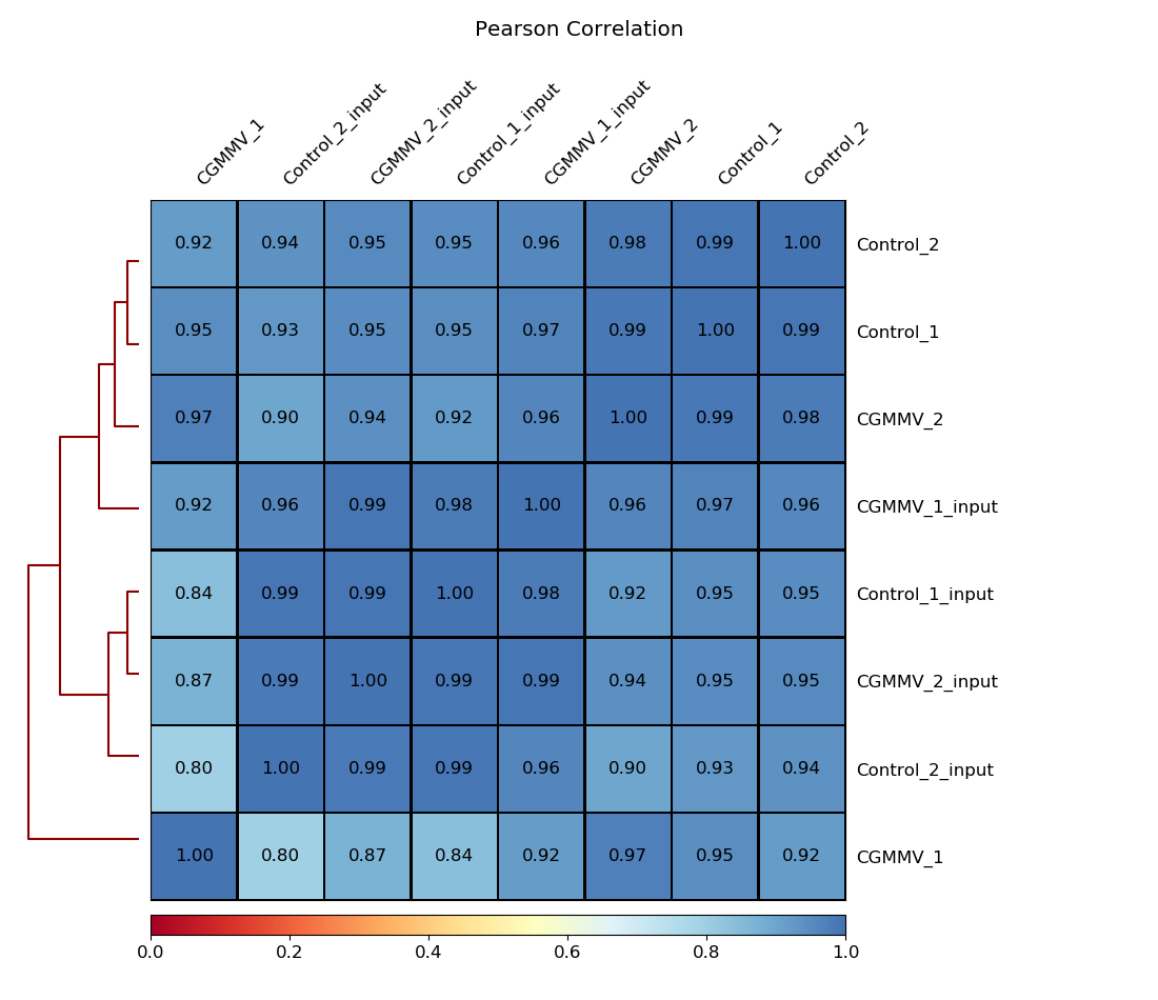

Supplement: Supplementary file 2 — Additional file 2: Supplementary file 2 Figure S2. Gene ontology (GO) analysis (GO: level 2) of the m6A-containing transcripts identified in m6A -seq in watermelon. [file 12870_2021_3289_MOESM2_ESM.png]

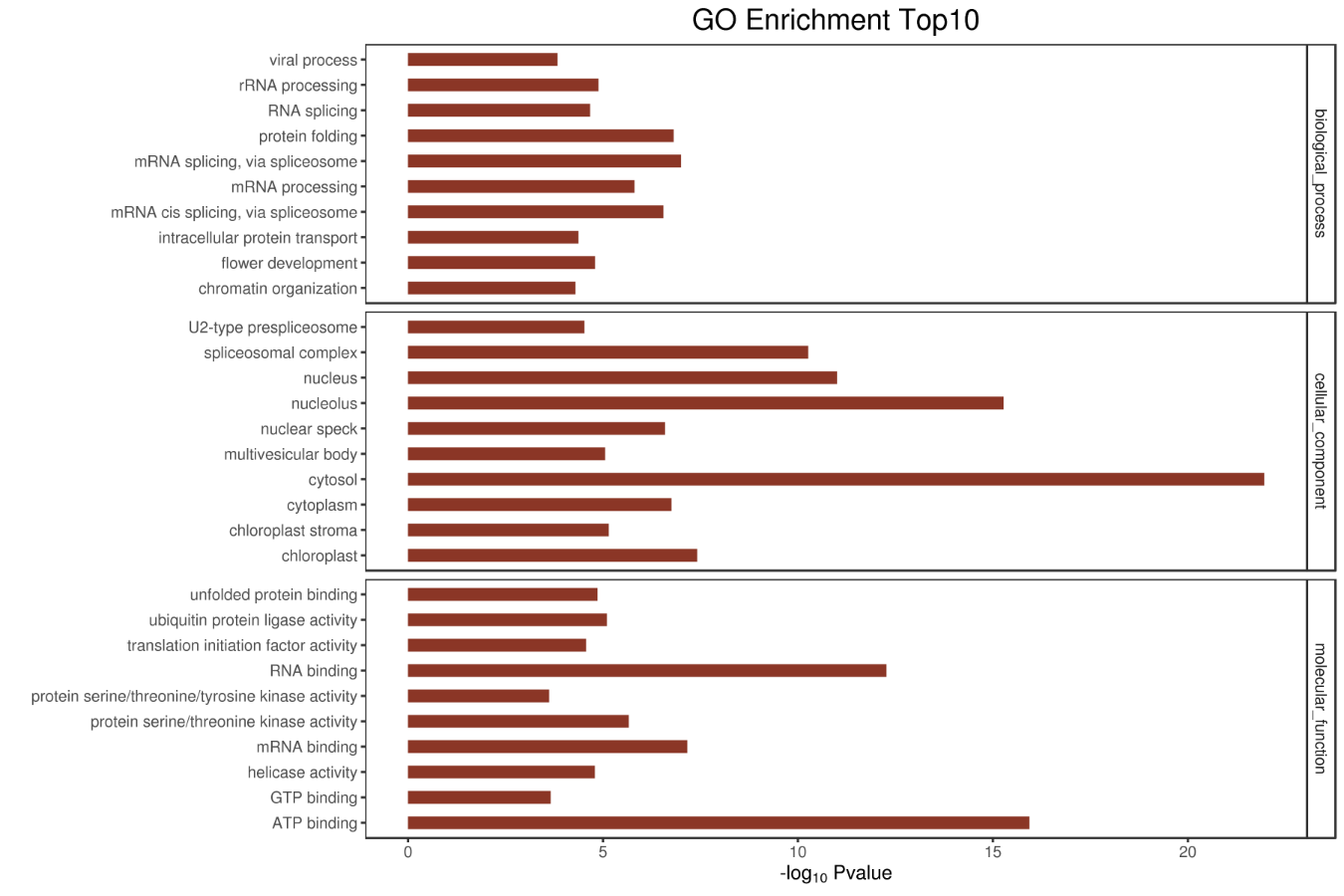

Supplement: Supplementary file 3 — Additional file 3: Supplementary file 3 Figure S3. Pie charts depicting the fraction of DMPs in four non-overlapping transcript segments. [file 12870_2021_3289_MOESM3_ESM.png]

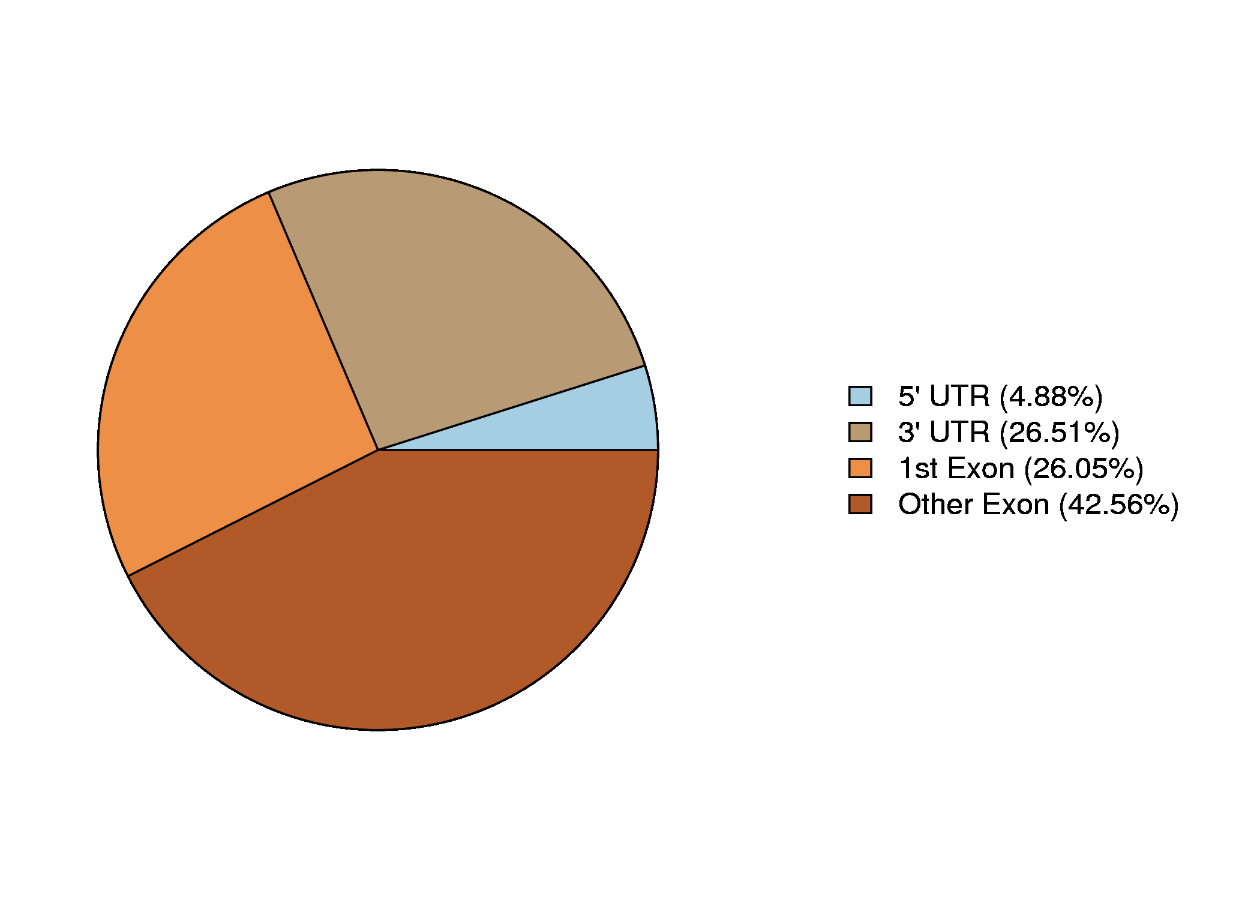

Supplement: Supplementary file 4 — Additional file 4: Supplementary file 4 Figure S4. The phenotype of two watermelon germplasm (R1288 and S1252) plants infected CGMMV 20 d. [file 12870_2021_3289_MOESM4_ESM.png]
